# Localization of Motor Neurons and Central Pattern Generators for Motor Patterns Underlying Feeding Behavior in *Drosophila* Larvae

**DOI:** 10.1371/journal.pone.0135011

**Published:** 2015-08-07

**Authors:** Sebastian Hückesfeld, Andreas Schoofs, Philipp Schlegel, Anton Miroschnikow, Michael J. Pankratz

**Affiliations:** LIMES-Institute, University of Bonn, 53115, Bonn, Germany; Columbia University, UNITED STATES

## Abstract

Motor systems can be functionally organized into effector organs (muscles and glands), the motor neurons, central pattern generators (CPG) and higher control centers of the brain. Using genetic and electrophysiological methods, we have begun to deconstruct the motor system driving *Drosophila* larval feeding behavior into its component parts. In this paper, we identify distinct clusters of motor neurons that execute head tilting, mouth hook movements, and pharyngeal pumping during larval feeding. This basic anatomical scaffold enabled the use of calcium-imaging to monitor the neural activity of motor neurons within the central nervous system (CNS) that drive food intake. Simultaneous nerve- and muscle-recordings demonstrate that the motor neurons innervate the cibarial dilator musculature (CDM) ipsi- and contra-laterally. By classical lesion experiments we localize a set of CPGs generating the neuronal pattern underlying feeding movements to the subesophageal zone (SEZ). Lesioning of higher brain centers decelerated all feeding-related motor patterns, whereas lesioning of ventral nerve cord (VNC) only affected the motor rhythm underlying pharyngeal pumping. These findings provide a basis for progressing upstream of the motor neurons to identify higher regulatory components of the feeding motor system.

## Introduction

Spatial and temporal execution of motor programs reflects the behavior of an organism, which results from the processing of external and internal information by the central nervous system. The most basic components of complex behaviors consist of repetitive, stereotyped movements, like breathing [1], walking, swimming, chewing, swallowing and crawling. Such stereotypical movements are often driven by CPGs, a network of neurons which has the intrinsic capability to produce a rhythmic neural activity that drives the final movement [2]. Our fundamental knowledge on the function of CPGs in the generation of rhythmic behavior derives from work in vertebrate and invertebrate model organisms over the last 40 years [3,4], like locomotion in lampreys, cats and stick insects [5–9] or the gastric mill/pyloric filter rhythm from the crustacean stomatogastric nervous system (STG)[10]. Strongest evidence for a CPG driving rhythmic behavior derived from studies in which isolated CNS were shown to be capable of generating motor patterns in the absence of extrinsic sensory input [11]. Such motor patterns, which are not fine tuned by external or internal signals, would reflect the basic components of a given behavior in the intact organism, have been termed fictive behavior [10].

Feeding behavior of *Drosophila melanogaster* larvae offers a unique opportunity to investigate the molecular and cellular basis of neuronal substrate that underlies rhythmic motor behaviors. The rhythmic feeding movements of the *Drosophila* larva have been broadly described [12–15], while the gross anatomy of the appropriate musculature and innervating nerves has been described in different Diptera larval species [16,17]. Fictive feeding behavior in *Drosophila* larva was also recently established, based on the motor patterns recorded from three pharyngeal nerves innervating the feeding related musculature [17].

Building on these studies, in which morphological and physiological analyses focused mainly on the periphery, we have been utilizing genetic and imaging tools [18,19] to investigate neural circuits underlying feeding behavior within the CNS of *Drosophila* larva. In a recent study, we have identified numerous populations of central neurons that modulate feeding motor programs [20]. In this study, we characterize the activity and the anatomy of distinct sets of glutamatergic neurons that comprise the motor neurons for food intake and feeding-related movements (i.e., pharyngeal, mouth hook and head movements). We show that CPGs for fictive feeding are located in the SEZ, and that the brain hemispheres are necessary to maintain appropriate motor patterns underlying feeding.

## Results

### Feeding muscles and nerves

**Fig 1:**
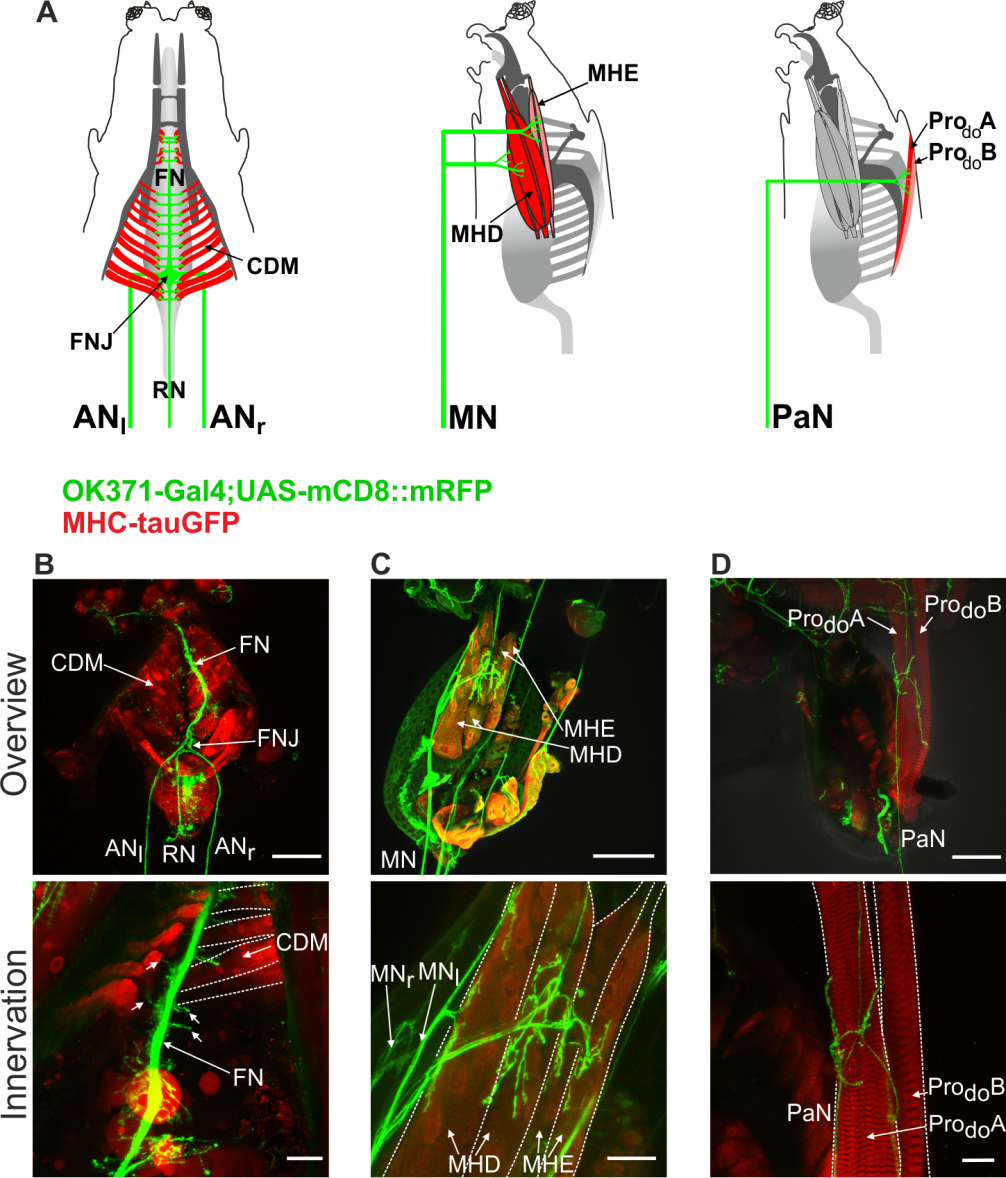
Peripheral innervation of glutamatergic axons onto feeding related muscles. **A**, Schematics of the innervation of antennal nerve (AN_l_ = left, AN_r_ = right) onto the cibarial dilator musculature (CDM), maxillary nerve (MN) onto mouth hook elevator (MHE) and mouth hook depressor (MHD) and prothoracic accessory nerve (PaN) onto prothoracic dorsal muscle A and B (Pro_do_ A/B). **B-D**, Overview of immunohistochemical staining of the larval CPS (genotype: OK371-Gal4/MHC-tauGFP;UAS-mCD8::mRFP/+) and innervation of the glutamatergic axons onto the muscles (top panels); Zoom of the neuromuscular junction of AN (fused to FN at the CDM), MN and PaN (**B,C,D,** respectively, bottom panels). FN, frontal nerve; FNJ, frontal nerve junction; RN, recurrent nerve. Scale bars: B,C,D upper panel: 100μm; B lower panel: 50μm; C,D lower panel: 25μm.

The muscles responsible for larval feeding cycle are innervated by three major pharyngeal nerves (Fig 1A; [17]). Pharyngeal pumping is mediated by the CDM, which is innervated by the antennal nerve (AN); mouth hook movements are mediated by mouth hook elevator (MHE) and mouth hook depressor (MHD) muscles, which are innervated by the maxillary nerve (MN); and head tilting is mediated by the dorsal protractor muscles A and B (Pro_do_A and Pro_do_B) that attach the head skeleton to the body wall, and are innervated by the prothoracic accessory nerve (PaN) [17]. We first wanted to determine the innervation patterns of the motor neuron axons onto the various muscles involved in the feeding movements. For this we used a Gal-4 driver line (OK371) that drove target gene expression in nearly all glutamatergic neurons of CNS, including the motor neurons [21], and MHC-tauGFP line (myosin heavy chain) to visualize the muscles [22].

The glutamatergic axons projecting through the AN fuse near the posterior end of the CDM at the frontal nerve junction (FNJ), from which the frontal nerve (FN) extends anteriorly in between the CDM (Fig 1B). Small neurites extend out from the axons onto the muscles. For the MN, neuromuscular junction could be visualized at the MHD and MHE muscles (Fig 1C). Innervation of the PaN onto Pro_do_A and Pro_do_B muscles is shown in Fig 1D. These results show that glutamatergic axons innervate the muscles involved in feeding. This is in accordance to previous studies showing that motor neurons in the VNC are also glutamatergic [21].

### Identification of glutamatergic neurons innervating the feeding muscles

**Fig 2:**
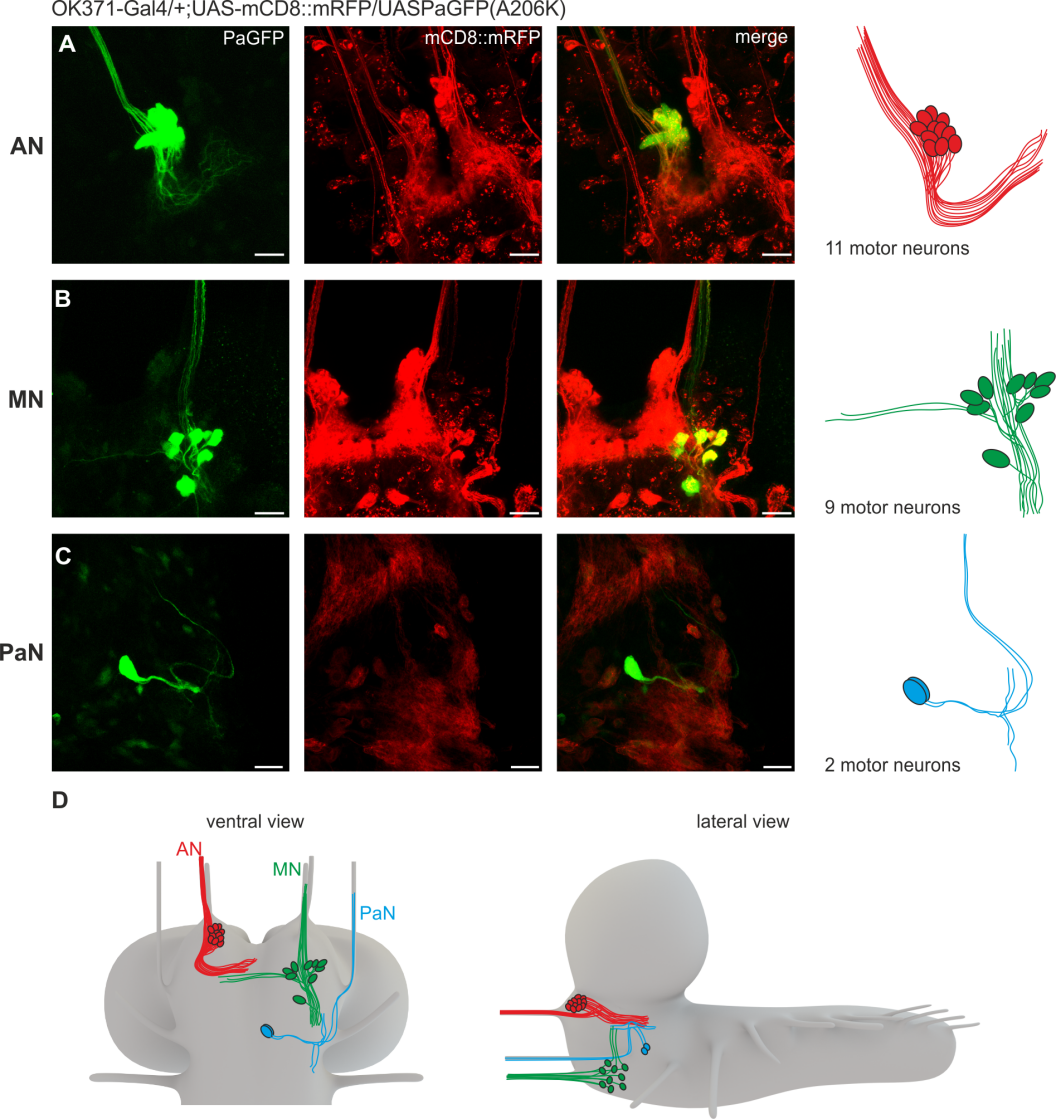
Anatomical identification of feeding related motor neurons using PaGFP. **A**, Glutamatergic neurons projecting through the antennal nerve (AN) visualized by activation of PaGFP (genotype: OK371-Gal4/UAS-mCD8::mRFP;UAS-PaGFP(A206K/+). Up to 11 motor neurons could be identified for the AN. Left = PaGFP, middle = mRFP, right = merge. **B**, Glutamatergic neurons projecting through the maxillary nerve (MN). Up to 9 motor neurons could be identified. **C**, Glutamatergic neurons projecting through the prothoracic accessory nerve (PaN). Two motor neurons could be identified. **D**, Schematic overview (ventral and lateral) of the identified glutamatergic neurons in the subesophageal zone (SEZ) projecting through the AN, MN and PaN. Scale bars: 20μm.

We next wanted to identify the motor neurons for all three pharyngeal nerves within the CNS. Our strategy was to use photoactivatable GFP (PaGFP) assisted circuit mapping with a two-photon laser [23,24], using OK371-Gal4 driving UAS-mCD8::mRFP;UAS-PaGFP(A206K). A small area of a pharyngeal nerve was activated and the diffusing GFP signal was traced back to the CNS. Somata in the SEZ, which showed enhanced green fluorescence after activation of the nerve, were subsequently photoactivated in order to increase fluorescence. This was performed for each of the three pharyngeal nerves (Fig 2).

Activation of PaGFP in the AN resulted in labeling of a tight cluster of up to 11 cells in the dorsal SEZ just lateral to the foramen (10.3 ± 0.95 cells, n = 10, Fig 2A), with dendritic arborizations in the anterior midline of the SEZ. This observation was further confirmed by retrograde nerve fillings with tetramethyrhodamine-dextran (Tmr-D) of the AN, which resulted in labelling of the CDM motor neuron cluster (colocalization with OK371-Gal4 driving UAS-GCamP6s). Up to two additional cells were labelled by Tmr-D, which did not colocalize with cells marked by OK371-Gal4 (S1A Fig and S1 File)(see Discussion for what these extra labelled cells could represent).

Activation of PaGFP in the MN resulted in labeling of a more loosely organized cluster with up to 9 cells at the ventro-lateral border of the SEZ (8.25 ± 0.7 cells, n = 8, Fig 2B). Several of the neurons projecting through the MN showed dendritic arborizations in the midline of the SEZ near the dendritic fields of the neurons labelled by the AN. Retrograde filling of the MN showed that 9 glutamatergic neurons were projecting out of the CNS via the MN (S1B Fig and S2 File).

Activation of PaGFP in the PaN resulted in the labeling of two cells located in medial SEZ (2 cells, n = 15, Fig 2C). Dye filling labelled additional two non glutamatergic cells projecting out of the CNS via the PaN (S1C Fig and S3 File)(see Discussion). Schematics of the relative locations of glutamatergic motor neurons in the CNS are shown in Fig 2D.

### Imaging rhythmic activity of pharyngeal nerves

**Fig 3:**
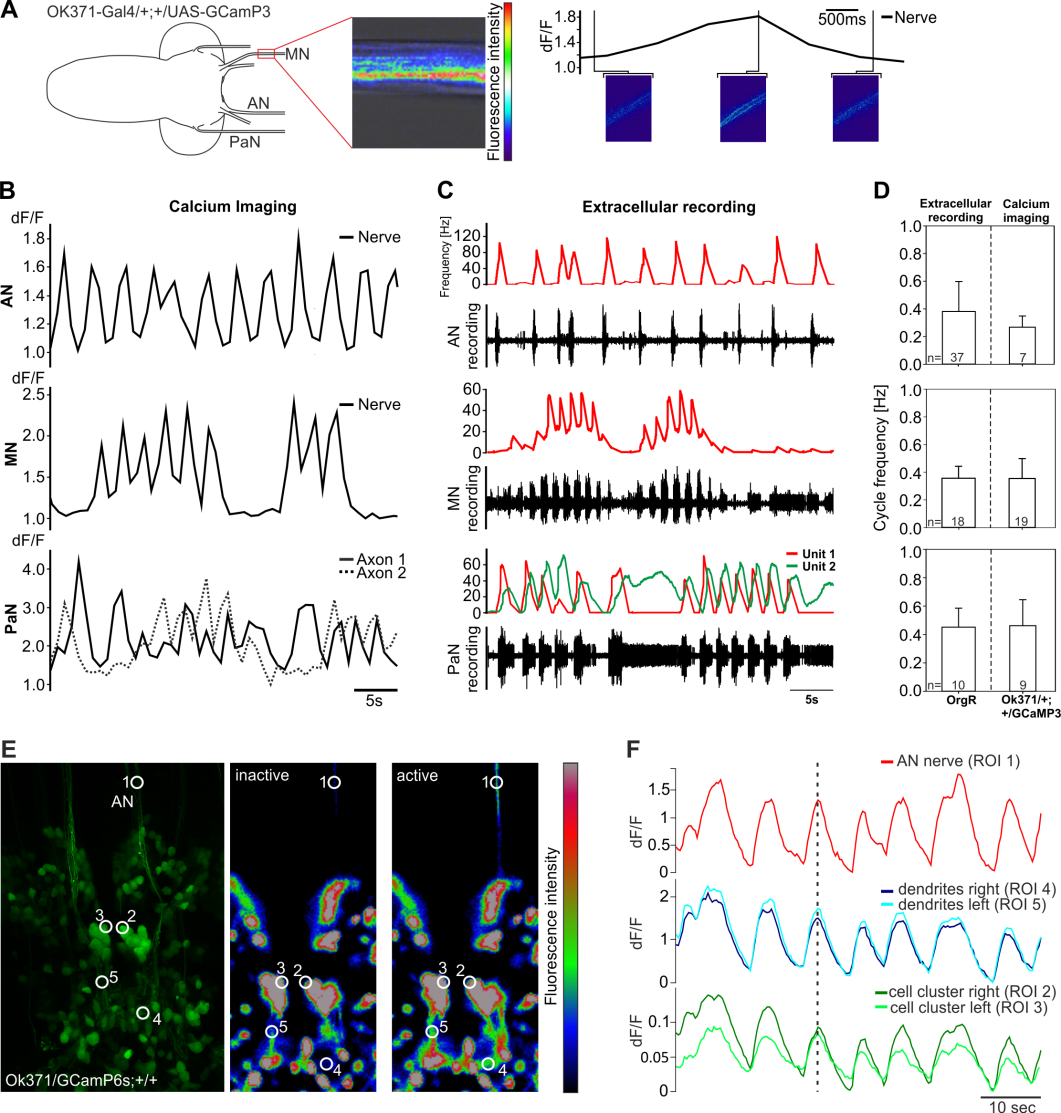
Comparison between calcium activity and extracellular recordings from AN, MN and PaN. **A**, Experimental setup for calcium imaging of the three pharyngeal nerves. Here shown for the MN. **B**, Calcium transients and **C**, extracellular recordings for all three pharyngeal nerves (AN, MN and PaN). Note the temporal correlation similarities between optogenetic and electrophysiological data. **D**, Quantitative comparison of measured cycle frequencies in all three nerves show no significant difference. **E**, left panel = maximum projection of the SEZ region in larvae with the genotype OK371-Gal4/UAS-GCamP6s;+/+, middle/right panel = location of AN, motor neurons and dendritic arborizations being inactive/active. White circles showing regions of interest (ROI) used for measurements shown in F. **F**, Calcium activity from AN on one side temporally correlates to the activity of AN motor neurons and their dendritic fields on ipsi- and contralateral sides. AN, antennal nerve; MN, maxillary nerve; PaN, prothoracic accessory nerve. dF/F = change of fluorescence intensity over baseline fluorescence.

If the identified glutamatergic neurons comprise the motor neurons driving the different movements of feeding behavior, they should also be rhythmically active. We first asked whether the axons were rhythmically active, thus reflecting the motor pattern recorded from the pharyngeal nerves. Calcium indicators can be utilized to measure neuronal activity as a complement to electrophysiological methods, since action potentials and the induced synaptic transmissions generate large and rapid cytoplasmic [Ca^2+^]-transients. Therefore, we expressed the genetically encoded calcium indicator GCaMP3 [25] in glutamatergic neurons using the OK371 driver line, and measured the neuronal activity of the three pharyngeal nerves (Fig 3A). In all cases, calcium imaging analysis revealed spontaneous, rhythmic activity (Fig 3B).

Comparison of bursting activity from extracellular recordings and calcium-imaging data (Fig 3C) revealed close temporal correlation of the rhythmic motor patterns observed in the AN, MN and PaN (performed Mann-Whitney-Rank-Sum-Test revealed no significant differences; Fig 3D). Taken together, calcium imaging analysis showed that the glutamatergic motor neurons display rhythmic activity, in correlation with the rhythmic motor output recorded extracellularly from the respective nerves.

We next wanted to determine the neural activity of the previously identified neurons, focusing on the CDM motor neurons that project through the AN. By expressing a modified version of the previously described calcium indicator (GCaMP6s [26], for enhancement of the signal to noise ratio), we observed temporal correlation of calcium activity between the AN, the two clusters of CDM motor neurons and their dendritic arborizations in the SEZ (Fig 3E and 3F). These results suggested that these clusters house the somata of glutamatergic motor neurons that extend through the AN to innervate the CDM. Furthermore the calcium imaging results provide a rhythmic blueprint for larval pharyngeal pumping in the CNS.

### AN targets both ipsi- and contra-lateral muscle systems

Of the three nerves, the AN is the most dedicated to feeding behavior since it innervates the muscles that drive pharyngeal pumping. This nerve is also anatomically distinct, since it fuses together at the FNJ and projects as a single fiber, the FN, in between the muscle palisades (see Fig 1A). This is in contrast to either MN or PaN, both of which end in a bilateral fashion on either side of the pharynx. This led us to ask whether each AN (or axons in the AN) innervates CDM on both sides.

**Fig 4:**
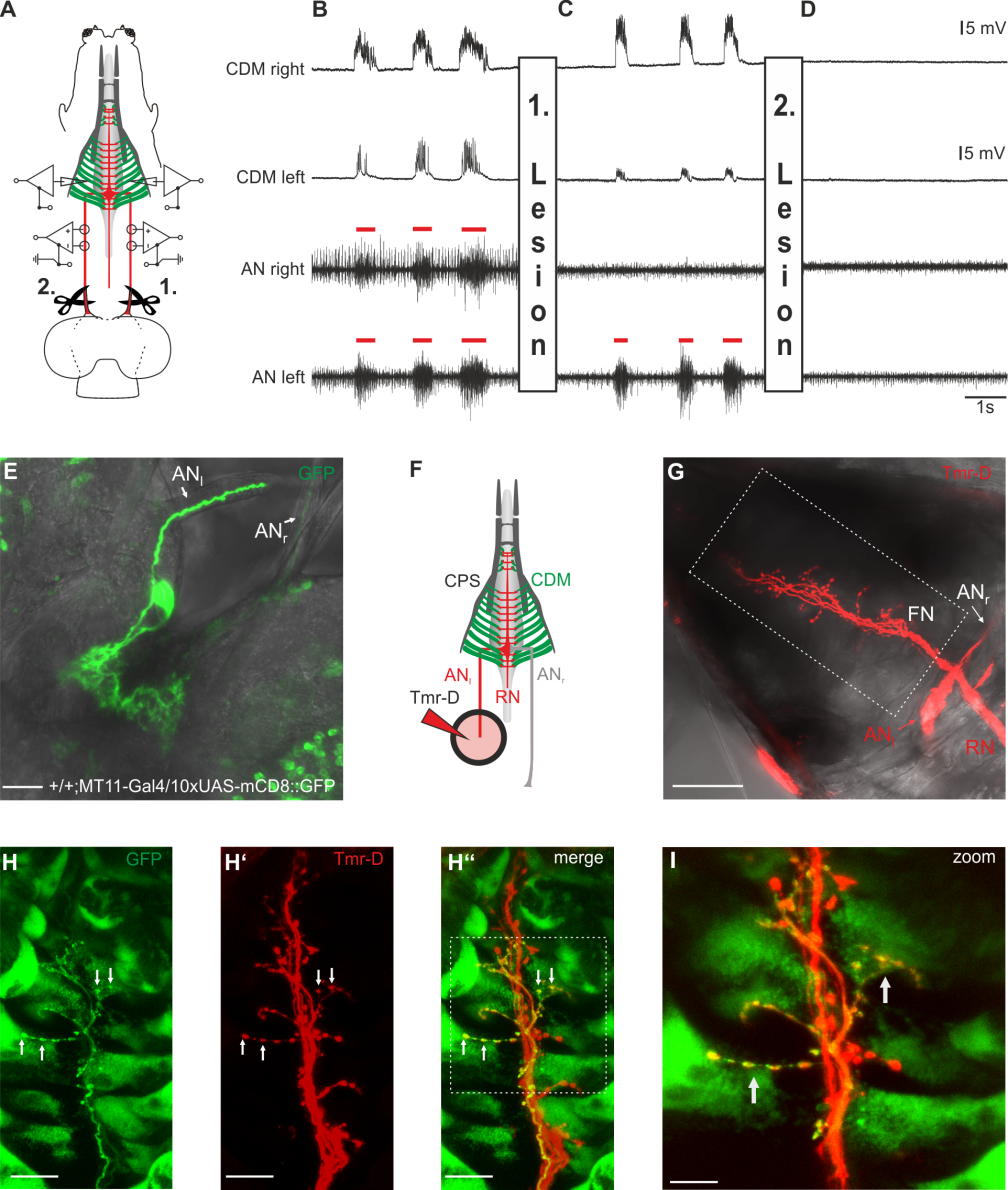
Motor neurons for pharyngeal pumping innervate the cibarial dilator musculature (CDM) ipsi- and contra-laterally. **A,** Simultaneous antennal nerve (AN) and cibarial dilator muscle (CDM) recordings of left and right side of the larval body of an OregonR larva. **B,** Under unimpaired conditions, AN motor pattern (red bars) of the left and right side is synchronous, and shows temporal correlation with the evoked postsynaptic potentials (PSPs) of the left and right CDM. **C,** Lesion of the right AN (1. Lesion) between the CNS and recording site results in abolishment of the corresponding AN motor pattern, whereas the PSPs on both sides of the CDM persist. Note that the diminished amplitude of the PSPs in the left CDM recording is caused by displacement of the glass electrode due to the lesion. **D,** Subsequent lesion of the left AN (2. Lesion) eliminated additionally the AN motor pattern on the left side and resulted in total disappearance of the PSP in left and right CDM. **E**, One CDM motorneuron (left side) strongly labelled in a larvae expressing 10xUAS-mCD8::GFP driven by MT11-Gal4. **F**, Schematic of the setup used for anterograde filling of the AN with tetramethylrhodamine-Dextran (Tmr-D). **G**, Tmr-D filled left AN shows axons with bilateral innervation of the CDM. **H-H”**, Colocalization of the Tmr-D labelled FN and one Axon of MT11-Gal4 driving 10xUAS-mCD8::GFP showing bilateral innervation of the CDM by one CDM motor neuron. **I**, Magnified region of the FN (marked in H” by white dotted box). Scalebars: E: 20μm,G: 50μm, H-H”: 20μm, I:10μm.

Therefore, we performed a series of lesion experiments together with simultaneous recordings from both nerves (extracellularly) and muscles (intracellularly) (see Fig 4A for experimental scheme); this setup thus represents a quadruple recording. In the unimpaired state (Fig 4B), both ANs show a simultaneous motor pattern that temporally correlates with the postsynaptic potentials (PSPs) in the CDM recordings. After completion of the first lesion on the right AN (see 1.in Fig 4A), no neural activity is detectable in the right AN, whereas the motor pattern in the left AN still persists, as expected. Remarkably, however, both left and right CDM show series of PSPs which are elicited by the motor pattern of the remaining left AN (Fig 4C). As a control, a second lesion was performed by cutting the left AN (both ANs are now cut), which abolished its motor pattern and the muscle activity in both CDMs (Fig 4D). These observations indicate that the CDM motor neurons on one side of the CNS innervate the muscles ipsi- and contra-laterally. Subsequently we used the published MT11-Gal4 line, which drives expression in one CDM motor neuron per side in the larval CNS [27]. In larvae carrying MT11-Gal4 driving 10xUAS-mCD8::GFP we observed that GFP expression strength varies in individual larvae (Fig 4E). We used this advantage to use a CNS which shows strong GFP expression in one CDM motor neuron and subsequently backfilled the AN nerve with tetramethylrhodamine-Dextran (Tmr-D) to visualize the potential bilateral innervation of the CDM (Fig 4F and 4G). By labelling exclusively the left AN, we could see a bilateral innervation onto the CDM muscles by the visualized axons. Colocalization with the MT11-Gal4 line driving 10xUAS-mCD8::GFP shows that one CDM motoneuron projects onto both, the ipsi- and contralateral side of the CDM (Fig 4H–4I).

### Manipulation of glutamatergic motor neuronal activity

**Fig 5:**
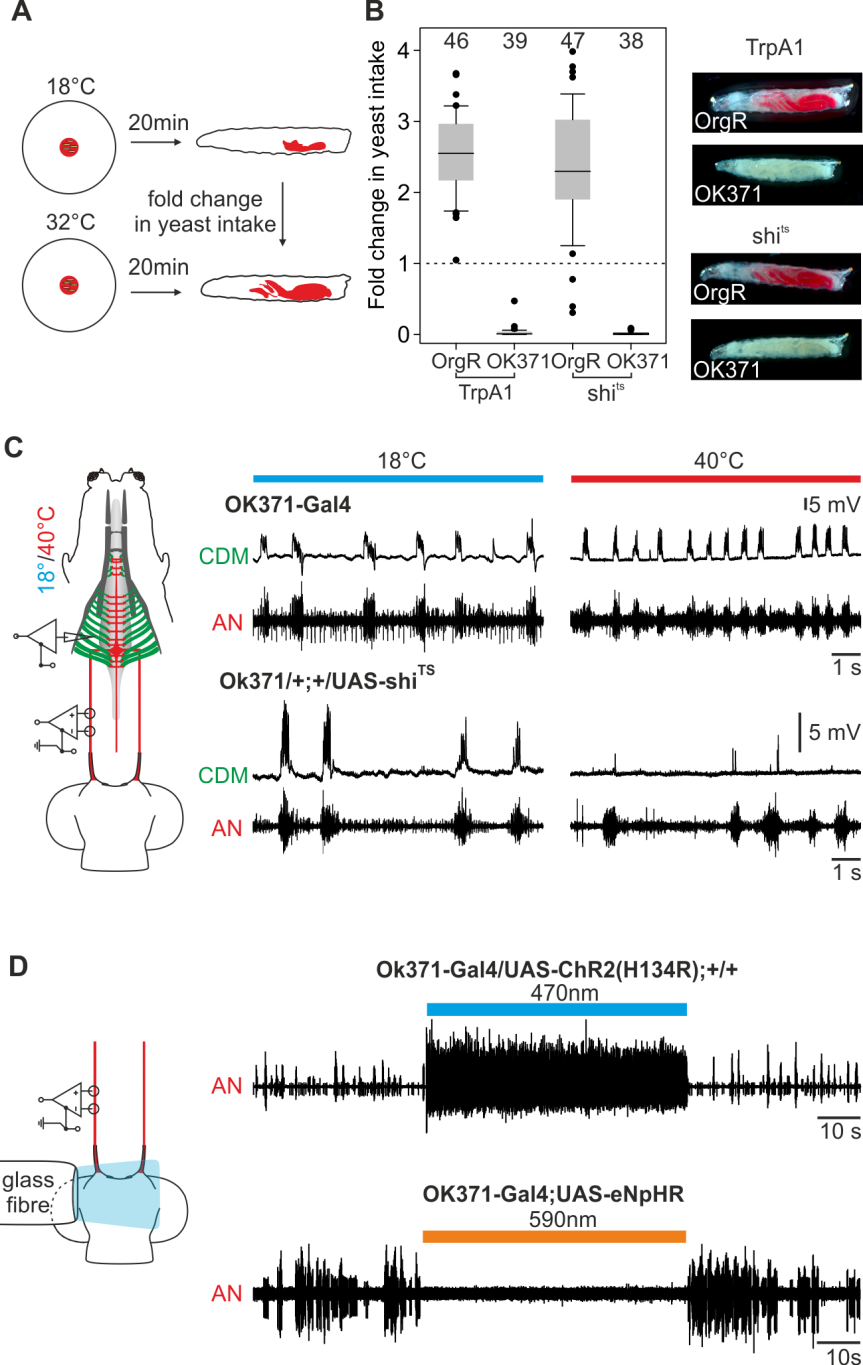
Activation and inactivation of feeding related motor neurons leads to decline in feeding. **A**, Experimental setup: yeast intake of larvae at 18°C and 32°C (% of larval body stained with red yeast) was determined after 20min of TrpA1-activation and shi^TS^-inactivation. **B**, Fold change in yeast intake; in both cases no dyed food could be observed in experimental larvae. **C**, Inactivation by shi^TS^ of glutamatergic neurons results in decline of cibarial dilator muscle (CDM) postsynaptic potentials (PSP), but not in the antennal nerve (AN) motor pattern. **D**, Activation of glutamatergic neurons via channelrhodopsin (UAS-ChR2(H134R) or inhibition via halorhodopsin (UAS-eNpHR) resulted in direct tonic excitation or complete inhibition of the AN motor pattern.

The line OK371-Gal4 labels a large population of motor neurons, if not all, and neuronal activation using this line results in a tonic excitation pattern of AN and other pharyngeal nerves [20]. Since this Gal4-line also marks the feeding motor neurons, inhibiting OK371-Gal4 labelled neurons should lead to a decrease in food intake and an inhibition of muscle contraction. Therefore, we expressed shibire^TS^ in glutamatergic neurons (OK371-Gal4 drives UAS- shibire^TS^), which blocks synaptic transmission upon shifting to restrictive temperature, and performed food intake assays as well as recording extracellularly from the AN and intracellularly from the CDM (Fig 5A–5C). We focused on the AN/CDM pair since it was technically more feasible to perform double recordings from the nerve and the innervated muscles.

Inhibiting glutamatergic neurons completely eliminated food intake (Fig 5A and 5B). This was similar to what was observed when these neurons are activated through TrpA1(OK371-Gal4 driving UAS-TrpA1) (Fig 5B; [20]). Since this inactivation through shibire^TS^ and the activation through TrpA1 target a large population of glutamatergic neurons, we also performed simultaneous nerve (AN) and muscle (CDM) recordings. Inactivation through shibire^TS^ by temperature shift to 40°C near the CPS did not affect the motor pattern of the AN. By contrast, CDM recordings showed no post-synaptic potentials as a response to AN motor output (Fig 5C), indicating that the motor neurons for pharyngeal pumping are inactivated by shibire^TS^. Only a few single post-synaptic potentials with decreased peak topeak amplitude were detectable upon blocking the synaptic transmission by shibire^TS^. Therefore shibire^TS^ is able to block synaptic transmission in glutamatergic motor neurons projecting through the AN, which leads to physiological and behavioral alterations. These results were further confirmed by using light instead of temperature as manipulators of neuronal activity. Fig 5D shows that channelrhodopsin (UAS-ChR2(H134R)) mediated activation of glutamatergic neurons results in tonic excitation, whereas inhibition by halorhodopsin (UAS-eNpHR) leads to suppression of neural activity.

### CPG for feeding motor patterns is located in the SEZ

**Fig 6:**
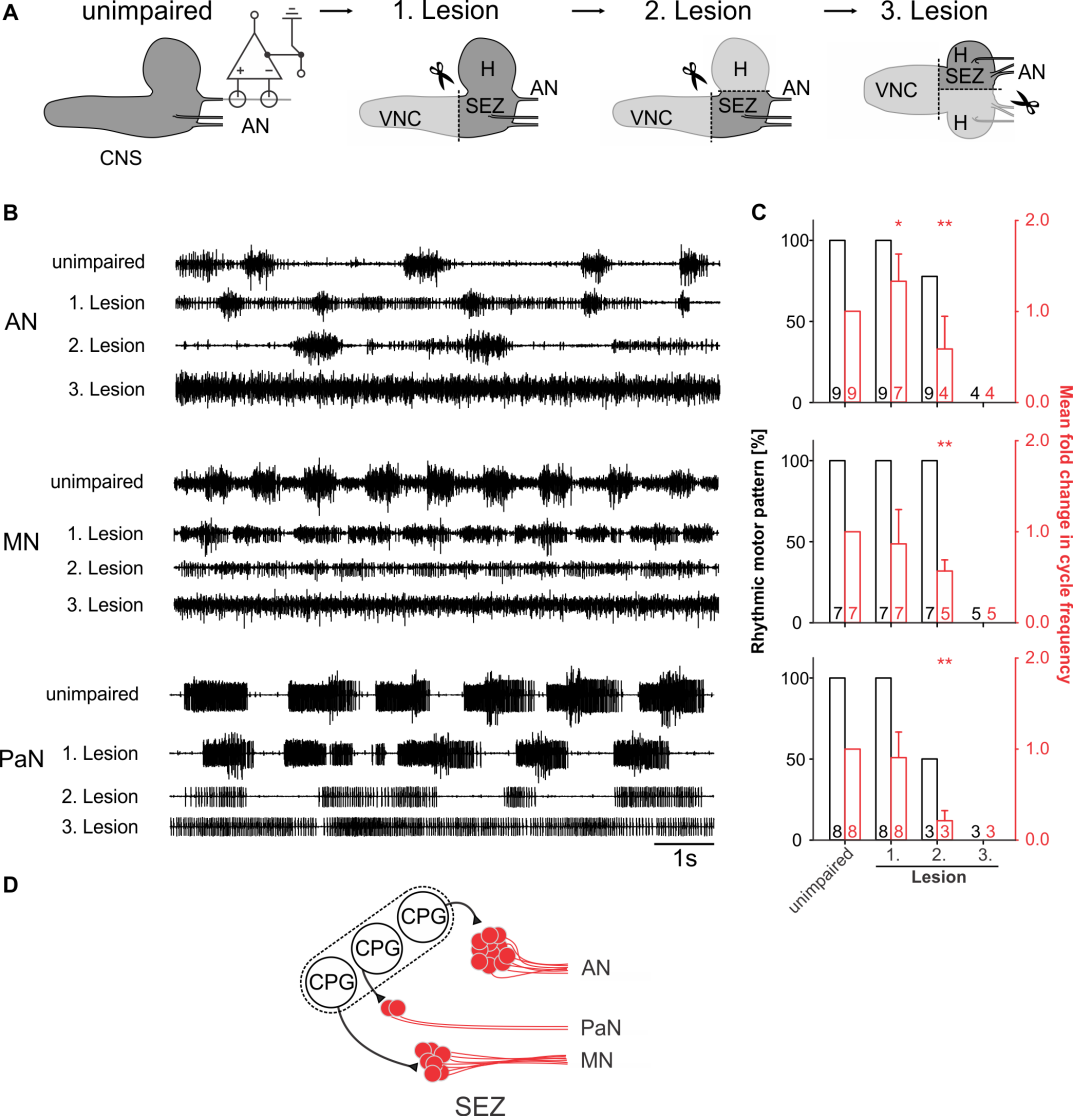
Central pattern generators (CPGs) of feeding-related motor patterns are localized to the subesophageal zone (SEZ). **A,** Starting with the intact central nervous system (CNS, unimpaired), antennal (AN), maxillary (MN) and prothoracic accessory nerve (PaN) motor patterns were individually recorded while successively lesioning the ventral nerve cord (VNC, 1. Lesion), brain hemispheres (H, 2. Lesion) and finally bisecting the SEZ (3. Lesion) of OregonR larvae. **B,** Single nerve recordings of AN, MN and PaN under unimpaired conditions, after consecutive removal of the VNC, hemisphere (H) and bisection of the SEZ. Note that the lesion of the VNC has minor effects on the rhythmic motor patterns, whereas removing the H severely impaired the motor patterns of AN, MN and PaN. Bisection of the residual SEZ results in tonic motor activity. **C**, Bar plots represent the percentage of the nerve recordings showing rhythmic motor activity (black) and the fold change in cycle frequency of the motor patterns (red) in the unimpaired state and after the successive lesions (n = number of experiments). **D,** Schematic drawing of the potential functional organization of CPG(s) driving the feeding related motor patterns in the SEZ. SEZ: subesophageal zone.

After having characterized the activity and anatomy of the motor neurons, we next wanted to determine where the feeding CPG was localized. To this end, we caused lesions to the isolated CNS preparation and simultaneously recorded the motor pattern (Fig 6A). This was done individually for the AN, the MN and the PaN. Lesion of the VNC did not affect the maintenance of AN, MN and PaN motor patterns (Fig 6B, 1. Lesion); however, it did result in increased cycle frequency of the AN motor pattern, whereas the motor pattern for MN and PaN were unaffected (Fig 6B, right graphs). These findings indicate that in the unimpaired state the VNC inputs can decelerate the AN motor pattern.

In the second lesion, both brain hemispheres were removed from the residual CNS. The remaining isolated SEZ was capable of generating rhythmic motor patterns in all three nerves. These data support the view that for all three motor patterns the CPG is located in the SEZ of the larval CNS. However, rhythm analysis of the patterns showed a severe decrease in cycle frequency of all motor patterns (Fig 6B, 2. Lesion), pointing to the importance of higher brain center inputs for adequate modulation of the CPG. The third lesion, in which the isolated SEZ was bisected along the longitudinal body axis, resulted in long lasting tonic activity of the neuronal units in all nerves (Fig 6B, 3. Lesion). Example of lesions performed for extracellular recordings is shown for the AN in S2 Fig. Taken together the lesion experiments indicate that the CPGs for feeding rhythm generation are localized in the SEZ (Fig 6D).

## Discussion

### Feeding motor neurons in the SEZ

Fundamental component of any motor system are the motor neurons that innervate the effector muscles. Our findings fill a crucial gap in knowledge by characterizing the motor neurons of the *Drosophila* larval feeding motor system. Cell bodies of the glutamatergic neurons that project through the three major pharyngeal nerves form spatially distinct clusters in the SEZ. Glutamatergic neurons projecting through the AN (which innervates the pharyngeal muscles) form a tight cluster of up to 11 cells. Additional cells, which do not colocalize with glutamatergic cells labelled by the OK371-Gal4 line could be visualized with retrograde filling of the AN. These are likely serotonergic cells, which are located close to the CDM motor neuron cluster as previously shown [28,29]. Neurons projecting through the MN (innervates the mouth hook muscles) form a more loosely organized cluster of 9 cells colocalizing with OK371-Gal4. No additional cells were labelled using retrograde fillings of the nerve. This indicates that neurons projecting out of the CNS via the MN are exclusively glutamatergic. Some of these may correspond to those identified by dye filling or lacZ enhancer trap staining of one of the pharyngeal nerves [27,30]. For the PaN (innervates muscles enabling head tilting), two glutamatergic neurons were found. Two additional neurons were labelled using retrograde filling. These neurons correspond to the two Hugin positive neurons on each side of the CNS that project out of the PaN. This is also consistent with two neuronal motor units described for PaN nerve recordings [17].

The identification of the motor neurons also enabled the monitoring of neuronal activity by calcium imaging analysis for one of these, the AN. This revealed that the rhythmic activity of the glutamatergic axonal projections and the rhythmic activity of the corresponding cell clusters in the SEZ are coincident. In addition, correlation of calcium imaging with electrophysiological recordings provides strong indication that the rhythmically active cells represent the respective motor neurons innervating the feeding muscles. A further notable feature of the AN is that its motor neurons innervate the CDM both ipsi- and contra-laterally. This might assure that the bilateral parts of the CDM operate in unison, a feature that would facilitate food intake.

### Feeding CPGs

A core element of behavior is built up of stereotypical repetitive movements controlled by CPGs which are embedded into a complex network of higher order neural circuits [31]. The cellular components that comprise a CPG have been characterized only in a few cases. For invertebrates, this includes the stomatogastric nervous system in crabs [32], food intake in *Lymnaea* [33], and locomotion in leech [34,35]. For vertebrates, only single components of CPGs have been described functionally [36,37].

In *Drosophila*, previous investigations showed, using surgical and genetic techniques, that neuronal circuits for the generation of larval crawling behavior are located in thoracic and abdominal segments of the CNS [38]. Furthermore they were able to show that crawling behavior is partially maintained directly after lesion of the brain hemispheres and SEZ and showed no difference in forward or backward peristaltic waves after several minutes. Although we do not know the cellular composition of the feeding CPGs, our lesion experiments demonstrate that they are localized in the SEZ and that for maintenance of a basic feeding rhythmic activity the brain hemispheres and the VNC are not necessary. Interestingly, generation of the rhythmic pattern requires interaction between the left and right side of the SEZ, since bisection of the SEZ leads to tonic firing of all three nerves. This result points to several possibilities, e.g., that interaction between both halves of the SEZ is essential for proper functioning of feeding CPG, that the lesion has injured the CPG and/or motor neurons in the SEZ. Future efforts will be aimed towards identifying specific Gal-4 lines targeting the different pharyngeal motor neurons and specific components of the feeding CPG, that will allow genetic manipulations of the brain hemispheres and the SEZ alone. The current study provides a foundation for further elucidation of the neural network underlying feeding in *Drosophila* larvae. Our identification of the motor neurons responsible for feeding movements will facilitate the identification of upstream cellular elements that comprise the CPGs and higher modulatory centers in the larval CNS.

## Materials and Methods

### Fly lines

Following lines were used: OK371-Gal4 (genotype w^1118^;OK371-Gal4;+, Bloomington #26160) for targeting glutamatergic neurons, MHC-tauGFP [22] as a muscle reporter line, MT11-Gal4 (genotype w;+;P{GawB}MT11)(Bloomington #37295), UAS-mCD8::mRFP (genotype y,w;UAS-mCD8::mRFP;+, Bloomington #27398 and y,w;+;UAS-mCD8::mRFP) (Bloomington #27399 (3^rd^ Chr.)),10xUAS-mCD8::GFP(genotype w;+;10xUAS-mCD8::GFP)(Bloomington #32184) UAS-TrpA1 (genotype w;UAS-TrpA1;+) (Bloomington #26263), UAS-shi^TS^ (w;+;UAS-shi^TS^ [39]), UAS-ChR2(H134R) (w;UAS-ChR2(H134R);+, Bloomington #28995), UAS-eNpHR (w;+;UAS-eNpHR::YFP, Bloomington #41752 gift from Leslie Griffith), UAS-PaGFP(A206K) (w;+;UAS-PaGFP(A206K), gift from A.S. Chiang [40]), UAS-GCamP3 (genotype w;+;UAS-GCamP3, Bloomington #32236), UAS-GCamP6s (genotype w^1118^;UAS-GCamP6s;+, Bloomington #42746), OregonR (genotype +/w;+/UAS-TrpA1;+/+). For PaGFP experiments genotype was y,w/w;UAS-mCD8::mRFP; UAS-PaGFP(A206K).

### Fly care

Flies and larvae were kept on 25°C under 12h light/dark conditions unless otherwise stated. 4h egg collections were made on apple juice agar plates containing a spot of yeast-water paste. After 48h, larvae were transferred into food vials containing lab standard fly food. All larvae used for the experiments were 98 +/- 2 h old. Only feeding larvae were used for the experiments.

For experiments with OK371-Gal4 driving either UAS-H134R-ChR2 or UAS-eNpHR.YFP, larvae were transferred after 48h into food vials containing additionally 100μM all-trans retinal (ATR). Food vials were darkened with surrounding aluminum foil to prevent ATR degradation.

### Electrophysiology

Extracellular recordings of pharyngeal nerves (AN, MN and PaN) and intracellular recordings of the CDM were performed as previously described in [20]. In brief, for extracellular recordings of pharyngeal nerves, each nerve was electrically isolated using a petroleum jelly pool surrounding the nerve placed on a piece of Parafilm. Silver wire electrodes were used for measuring with differential recordings motor output of the deafferented nerve. A preamplifier (Model MA103, Ansgar Büschges group electronics lab) connected to a four-channel amplifier/signal conditioner (Model MA 102, Ansgar Büschges group electronics lab) was used. All recorded signals were amplified (amplification factor: 5000) and filtered (bandpass: 0.1–3 kHz). Recordings were sampled at 20 kHz. Data was acquired with Micro3 1401 or Power 1401 mk2 A/D board (Cambridge Electronic Design) and Spike2 software (Cambridge ElectronicDesign). Intracellular muscle recordings of the CDM were recorded using glass microelectrodes filled with 3 M KCl solution (tip resistance: 20–30 MΩ) connected to an intracellular amplifier (BRAMP-01R, npi electronic GmbH). All recordings were digitally sampled by a Power 1401 mk2 A/D board (Cambridge Electronic Design) at 20 kHz. Data was acquired with Spike2 software (Cambridge Electronic Design).

### Temperature stimulation

Application of temperature shifts to the CNS was accomplished using a custom built temperature stimulator as described earlier in [20]. For shibire^TS^ experiments (Fig 5C) the temperature stimulator was placed near the cephalopharyngeal skeleton (CPS) and heated up to 40°C to reach the permissive temperature at the CDM.

### Light stimulation

Mounted ultra-bright blue (470nm)/orange (590nm) LEDs (M470L2 and M590L2, Thorlabs) with collimated lenses and heat sink were used in experiments with UAS-ChR2(H134R) or UAS-eNpHR. LEDs were positioned in a custom built holder, supplemented with an optical multimode fiber (AFS200/220Y Thorlabs). Distal end of the optical fiber was placed directly over the ventral side of the CNS. LEDs were controlled by a LED controller unit (LEDBB1 Thorlabs) and light stimuli were delivered using A/D board (CED). All light stimuli were applied using highest intensity of the controller at 1mA. The optical fiber had a length of approx. 90cm with a transmission efficiency of >99.8%/m. Experiments were performed under dark conditions.

### Immunohistochemistry

For the staining of peripheral anatomy (Fig 1) a line with the genotype OK371-Gal4;UAS-mCD8::mRFP was used and crossed into MHC-tauGFP line. Dissection protocol for extracellular recordings was used, leaving all three pharyngeal nerves and projections intact. Remaining cuticle surrounding the CPS was removed. The sample of remaining CNS, CPS and nerves of interest was stained using anti-chicken-GFP (1:500, Abcam plc) and anti-mouse-mRFP (1:500) as primary antibodies. In brief, samples were fixed for 60min in 4% PFA and the washed with 0.5 PBT (2x10min, 2x15min, 2x30min), then tissue was blocked using 0.5 PBT containing 5% goat-serum for 60min. Primary antibody was added and incubated overnight at 4°C. Samples were washed the next day with 0.1 PBT (2x10min, 2x15min, 2x30min) and 60min blocked with 0.1 PBT containing 5% goat serum. Secondary antibodies (anti-mouse-Cy3, 1:250 Jackson ImmunoResearch and anti-chicken-Alexa488, 1:250 Invitrogen) were added and the samples were incubated at 4°C overnight. After washing the samples the next day, they were immediately scanned using a Laser scanning microscope (Zeiss LSM 780) equipped with a Zeiss LCI “Plan-Neofluar” 25x/0.8 Imm Korr DIC M27 objective and a Zeiss “PlanNeofluar” 10x/0.3 objective.

### Antero-/Retrograde nerve fillings

Larvae were dissected as described for extracellular recordings. A jelly pool was used to isolate the nerve of interest. Saline level was lowered until the fluids inside and outside the jelly pool were separated from each other. The nerve was cut within the jelly pool and saline was replaced with tetramethylrhodamine-dextran solution (Tmr-D) (Life Technologies, 3000MW anionic at 10mg/ml in distilled water). The preparations were stored at room temperature for 3 hr. After uptake of the dye, preparations were fixed in 4% paraformaldehyde (PFA) for 40 min, washed in PBS and mounted in Mowiol. The CNS was scanned the following day using a Laser scanning microscope (Zeiss LSM 780) equipped with a Zeiss LCI “Plan-Neofluar” 25x/0.8 Imm Korr DIC M27 objective. For colocalization with glutamatergic neurons we used larvae with the genotype OK371-Gal4/+;UAS-GCamP6s/+. Using GCamP6s for scanning the CNS had the advantage that the strong fluorescent signal was evenly distributed throughout the cells and their dendritic arborizations.

After verification of expression of the CDM motor neurons in the CNS of larvae (+/+;MT11-Gal4/10xUAS-mCD8::GFP), anterograde fillings of the left AN were done using the same procedure as described for retrograde dye filling.

### Photoactivation of PaGFP

The CNS, CPS and attached pharyngeal nerves of larvae with the genotype OK371-Gal4/UAS-mCD8::mRFP;UAS-PaGFP(A206K)/+ were placed with ventral side down onto a Poly-L-lysine (Sigma Aldrich) coated cover slide and mounted upside down in Ringer solution. Images were acquired using a laser scanning microscope (Zeiss LSM 780) with a Zeiss LCI “Plan-Neofluar” 25x/0.8 Imm Korr DIC M27 objective. To photoactivate the GFP we used a Ti:Sapphire Chameleon Ultra II Laser (Coherent) tuned to 820nm. Laser power was set to 8% for activation of the GFP in nerves. Bleach period of 8s was sufficient to activate the GFP in axons of the nerves. This was repeated two times at different areas of the nerve. Following settings were used: image size at 161.31x161.31μm, resolution at 256x256, pixel dwell 2μs, speed 11 and region of interest (ROI) size at 140μm.

After successful activation of GFP in the nerve, low GFP signal in somas in the SEZ were visible. Those were subsequently activated using the same protocol, adjusting ROI sizes to somata. This procedure was done for AN, MN and PaN. Z-stacks were acquired using 488nm laser and 561 nm laser as excitatory sources. Fluorescence was collected using photomultiplier tubes after filtering using the MBS 488/561/633 emission filter.

### Calcium imaging

Third instar feeding larvae with the genotype OK371-Gal4/+;UAS-GCamP3/+ (Fig 3A and 3B) or OK371-Gal4/UAS-GCamP6s;+/+ (Fig 3E and 3F) were used for calcium imaging experiments.

Neural activity of the three pharyngeal nerves was studied using semi intact preparations as described above consisting of CNS, CPS and the nerves. Calcium imaging was performed with Laser scanning microscope (Zeiss LSM 780) equipped with ZEISS LCI "Plan-Neofluar" 25x/0.8 Imm Korr DIC M27 objective dipped into the saline solution (Fig 3A and 3B). In Fig 3E and 3F CNS with nerves and CPS was placed ventral side down onto a polylysine coated cover slide and imaged upside down to visualize somata and dendritic arborizations in the SEZ (scan speed: 347.7ms). For excitation an argon laser with a wavelength of 488 nm was used. Fluorescence was collected using photomultiplier tubes after filtering using the MBS 488/561/633 emission filter. Images were acquired using Zen. Data were analyzed using the software Zen 2011 (Zeiss) and Spike2.

### Feeding assay

Feeding assay was performed as previously described in [20]. In brief, 5 larvae were starved for 30min on water soaked filter paper and then placed onto a drop of yeast-water-paste in the middle of an apple-juice-agar plate. The plates were placed on either 18°C or 32°C in an incubator for 20min. Afterwards larvae were washed in hot water (60°C) and pictures were taken using an Axiocam. Values were calculated as percentage of larval body stained red for each temperature and the fold change in yeast intake was calculated. For experiments with shibire^TS^, 32°C experiments were performed following 1h incubation at 32°C to ensure that the storage of synaptic vesicles are emptied.

### Lesion experiments

In the lesion experiments the typical semi-intact preparation of third instar larvae was used (see section: Electrophysiology; for details: [20]). Lesion of nerves and parts of CNS (VNC, brain hemispheres and SEZ) were ablated by a micro-dissecting scissor (Fine Science Tools). For the successive lesions of different brain regions, extracellular recording of the AN was started five minutes after the ablation.

### Data analysis

Processing of electrophysiological recordings was performed with a modified script of Spike2 software (provided by Cambridge Electronic Design). Statistical analysis of the electrophysiological data was accomplished using SigmaPlot (Version 12.0). For significance test we used Mann-Whitney Rank Sum Test (* p≤0.05, ** p≤0.01 and *** p≤0.001).

Food intake analysis was performed as described in [20] using a custom script in Fiji for analysis. Calcium imaging data was processed in Zen2009 and Spike2 software.

## Supporting Information

S1 Fig Anatomical identification of feeding related motor neurons using retrograde dye filling of pharyngeal nerves. Glutamatergic cells and dendrites were visualized using OK371-Gal4 driving UAS-GCamP6s (GCamP6s used for scanning due to very high signal quality in live scans). Each pharyngeal nerve was filled with tetramethylrhodamine-dextran (Tmr-D) for 3h, subsequently fixed in PFA and directly scanned. **A**, Retrograde filling of the antennal nerve (AN) revealed up to 14 neurons labelled by Tmr-D and up to 11 colocalized with OK371-Gal4 driving UAS-GCamP6s. **B**, Retrograde filling of the maxillary nerve (MN) revealed 9 neurons labelled by Tmr-D and all colocalized with OK371-Gal4 driving UAS-GCamP6s.**C**, Retrograde filling of the prothoracic accessory nerve (PaN) revealed 4 labelled neurons by Tmr-D, 2 neurons colocalized with OK371-Gal4 driving UAS-GCamP6s. Scale bars: most left panels: 50μm, magnified regions: 20μm.
(TIF)

S2 Fig Motor patterns of the antennal nerve (AN) and subsequent applied lesions during extracellular recording. Starting with the intact central nervous system (CNS, unimpaired), the antennal nerve (AN) motor pattern was recorded while successively lesioning the ventral nerve cord (VNC, 1. Lesion), brain hemispheres (H, 2. Lesion) and finally bisecting the subesophageal zone (SEZ) (3. Lesion). **A,** Single nerve recording of AN in unimpaired conditions. **B**, Removal of the VNC leads to acceleration of the AN motor pattern (recording trace). Imaginal discs of the prothoracic nerve (ProN) served as landmark for the lesion. **C**, Removal of the brain hemispheres (H) leads to slight deceleration of the AN motor rhythm. Optic stalks (OS) served as residual tissue for grabbing the hemispheres with forceps to ensure more precise lesion. **D,** Bisection of the residual SEZ leads to tonic activity in the AN motor pattern and abolishment of rhythmic activity of the motor neurons. Remaining imaginal discs of ProN and Mesothoracic nerve (MeN) served as landmarks for proper lesions.
(TIF)

S1 File Retrograde filling of the AN. The AN was filled with tetramethyrhodamine-dextran (Tmr-D) in larvae with the genotype OK371-Gal4/UAS-GCamP6s;+/+. Apart from cells colocalizing with the OK371-Gal4 expression pattern, up to two additional cells were labelled by Tmr-D. These might be serotonergic cells, which form a cluster of four neurons leaving the CNS via the AN (reference for the SE0 cluster) and whose cell bodies lie in the same area as the CDM motor neurons described here.
(AVI)

S2 File Retrograde filling of the MN. The MN was filled with tetramethyrhodamine-dextran (Tmr-D) in larvae with the genotype OK371-Gal4/UAS-GCamP6s;+/+. All cells labelled bey Tmr-D colocalized with glutamatergic cells.
(AVI)

S3 File Retrograde filling of the PaN. The MN was filled with tetramethyrhodamine-dextran (Tmr-D) in larvae with the genotype OK371-Gal4/UAS-GCamP6s;+/+. Two neurons colocalized with glutamatergic neurons. Two additionals neurons were labelled by Tmr-D. The additional cells likely correspond to the previously published hugin positive neurons leaving the CNS via the PaN.
(AVI)
