# Supplementary figures and images for: Localization of Motor Neurons and Central Pattern Generators for Motor Patterns Underlying Feeding Behavior in Drosophila Larvae
Source: PLoS One. 2015 Aug 7;10(8):e0135011. doi: 10.1371/journal.pone.0135011 (PMC4529123; doi:10.1371/journal.pone.0135011)

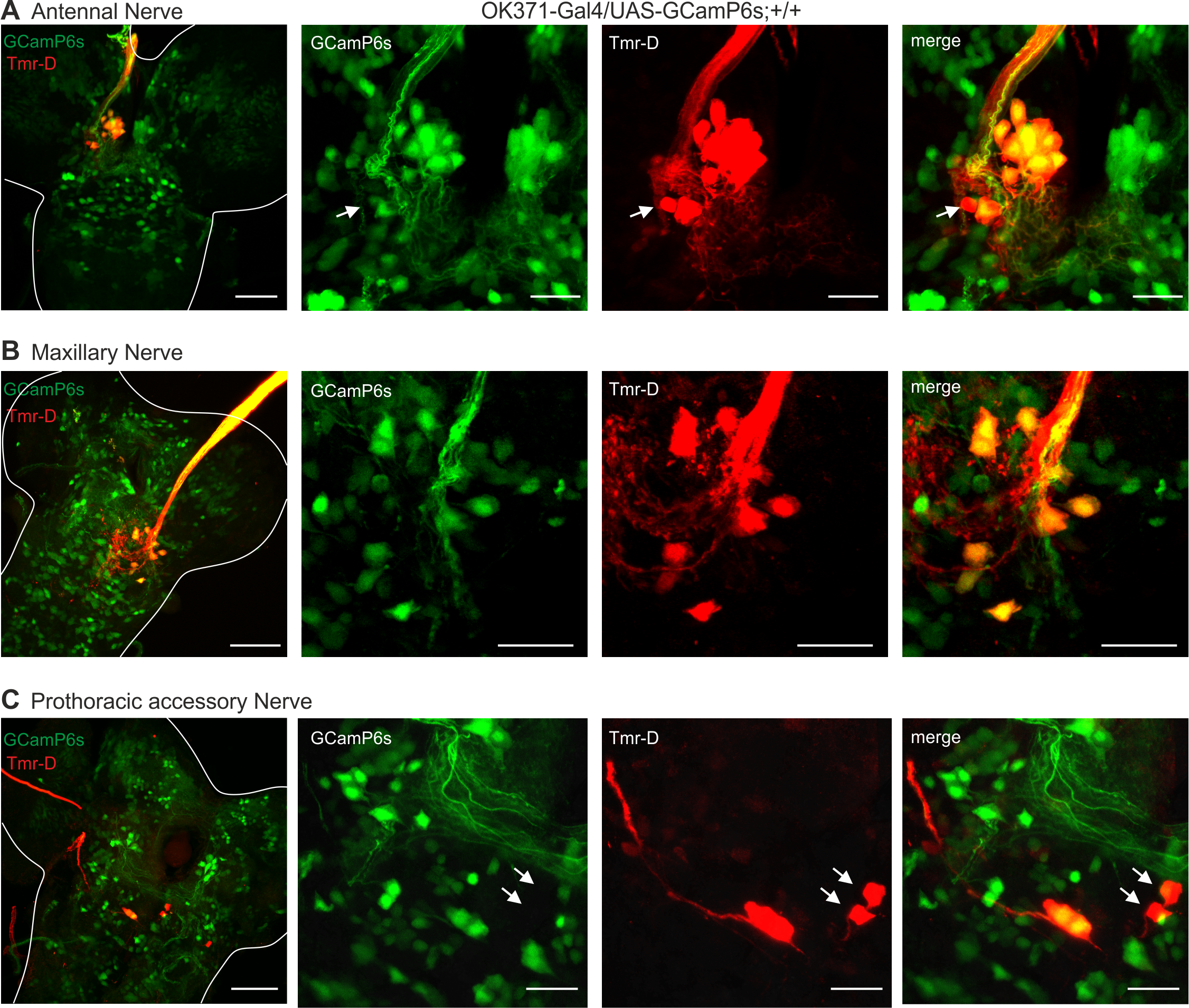

Supplement: S1 Fig — Glutamatergic cells and dendrites were visualized using OK371-Gal4 driving UAS-GCamP6s (GCamP6s used for scanning due to very high signal quality in live scans). Each pharyngeal nerve was filled with tetramethylrhodamine-dextran (Tmr-D) for 3h, subsequently fixed in PFA and directly scanned. A, Retrograde filling of the antennal nerve (AN) revealed up to 14 neurons labelled by Tmr-D and up to 11 colocalized with OK371-Gal4 driving UAS-GCamP6s. B, Retrograde filling of the maxillary nerve (MN) revealed 9 neurons labelled by Tmr-D and all colocalized with OK371-Gal4 driving UAS-GCamP6s.C, Retrograde filling of the prothoracic accessory nerve (PaN) revealed 4 labelled neurons by Tmr-D, 2 neurons colocalized with OK371-Gal4 driving UAS-GCamP6s. Scale bars: most left panels: 50μm, magnified regions: 20μm. (TIF) [file pone.0135011.s001.tif]

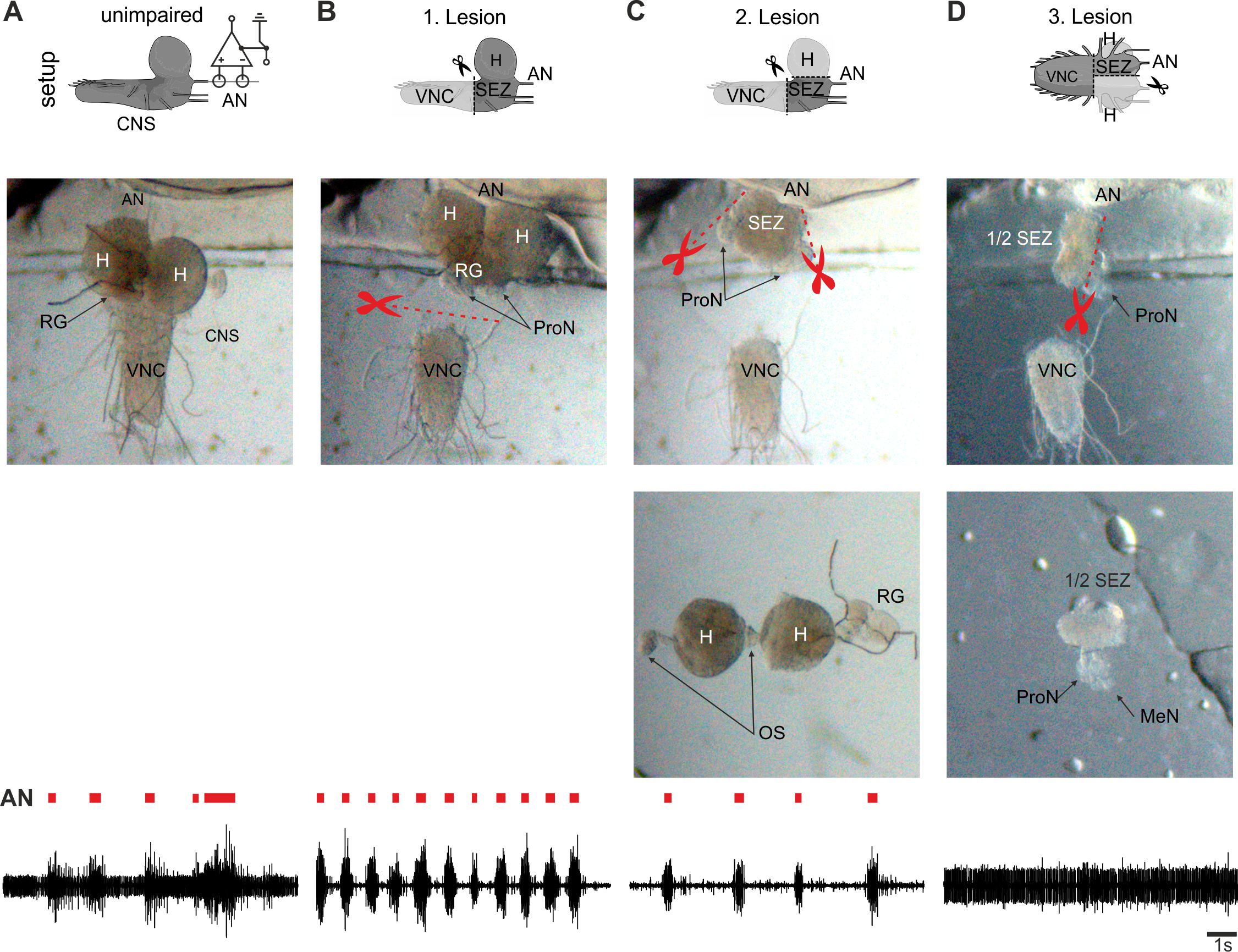

Supplement: S2 Fig — Starting with the intact central nervous system (CNS, unimpaired), the antennal nerve (AN) motor pattern was recorded while successively lesioning the ventral nerve cord (VNC, 1. Lesion), brain hemispheres (H, 2. Lesion) and finally bisecting the subesophageal zone (SEZ) (3. Lesion). A, Single nerve recording of AN in unimpaired conditions. B, Removal of the VNC leads to acceleration of the AN motor pattern (recording trace). Imaginal discs of the prothoracic nerve (ProN) served as landmark for the lesion. C, Removal of the brain hemispheres (H) leads to slight deceleration of the AN motor rhythm. Optic stalks (OS) served as residual tissue for grabbing the hemispheres with forceps to ensure more precise lesion. D, Bisection of the residual SEZ leads to tonic activity in the AN motor pattern and abolishment of rhythmic activity of the motor neurons. Remaining imaginal discs of ProN and Mesothoracic nerve (MeN) served as landmarks for proper lesions. (TIF) [file pone.0135011.s002.tif]
